# Assessment of Safe Listening Intentional Behavior Toward Personal Listening Devices in Young Adults

**DOI:** 10.3390/ijerph16173180

**Published:** 2019-08-31

**Authors:** Kamakshi V. Gopal, Sara Champlin, Bryce Phillips

**Affiliations:** 1 Department of Audiology and Speech Language Pathology, University of North Texas, Denton, TX 76203, USA; 2 Mayborn School of Journalism, University of North Texas, Denton, TX 76203, USA

**Keywords:** recreational noise, noise-induced hearing loss, theory of planned behavior, young adults, safe listening behaviors

## Abstract

Recreational noise-induced hearing loss (RNIHL) is a highly preventable disorder that is commonly seen in teenagers and young adults. Despite the documented negative effects of RNIHL, it is still challenging to persuade people to adopt safe listening behaviors. More research is needed to understand the underlying factors guiding listeners’ intentions to engage in safe listening habits. We used the Theory of Planned Behavior (TPB) to identify attitudes, social norms, and behavioral control in 92 young adults toward two intentional behaviors related to safe listening habits while listening to their personal listening devices: (1) lowering the intensity of loud music, and (2) shortening the listening duration of loud music. Using a Qualtrics survey, the major factors of the TPB model as they relate to the participants’ intention to engage in risk-controlling behavior were assessed. Behavioral intentions to turn the music down and listen for shorter durations were thought to be predicted by the TPB factors (attitudes, social norms, and perceived behavioral control). Linear regression findings indicated that the overall TPB models were significant. Positive attitudes toward turning the music down and shortening the durations were significantly associated with intentions to engage in non-risky behavior, more so for the former behavior.

## 1. Introduction

Recreational noise-induced hearing loss (RNIHL) is a common but highly preventable disorder that continues to be an important health concern, especially in teenagers and young adults. Much of this is due to increased use of unsafe listening habits in one or more professional or recreational activities, such as performing or practicing music, listening to loud music through a personal listening device (PLD), and frequent exposure to highly noisy places such as clubs, bars, concerts, music events, theaters, and sporting events. The World Health Organization (WHO) estimates that 1.1 billion young people worldwide could be at risk of hearing loss due to unsafe listening practices [1].

Several studies have shown that exposure to loud noise or music can lead to one or more auditory symptoms such as tinnitus, temporary hearing loss exhibited as temporary threshold shifts (TTS), permanent hearing loss exhibited as permanent threshold shifts (PTS), hyperacusis, recruitment, difficulty understanding speech, abnormal pitch perception, and aural fullness [2,3,4,5,6,7]. Hearing loss or sensitivity change is a result of the shift in hearing thresholds from excessive sound exposure. Recovery from the shift is based on the characteristics of exposure, including sound intensity, duration, and frequency, as well as individual differences/susceptibility among people being exposed [8]. If threshold shifts recover to the baseline, they are considered temporary or TTS. When exposure occurs at very high-intensity levels or is built up over time causes the thresholds to be worse than the baseline without ever recovering fully, it is referred to as PTS.

Excessive exposure to loud levels of sound can cause damage to the auditory pathway in any setting. Specific recommendations have been set for occupational noise exposure in the United States by both the National Institute for Occupational Safety and Health (NIOSH) and the Occupational Safety and Health Administration (OSHA) to help keep workers safe [9,10]. Current recommendations for both NIOSH and OSHA use a time-weighted average of a specific A-weighted decibel level (85 dBA for NIOSH and 90 dBA for OSHA) to indicate a 100% noise dose to compare with sound levels in a work setting. As the noise increases in intensity, the allowable exposure time to noise decreases to maintain the acceptable noise dose, thus preventing overexposure. No such recommendations or standards exist for recreational noise exposure. Currently, there are only loose guidelines in place to limit the output of PLDs and minimal recommendations on listening levels. This creates a risk factor as people continue to expose themselves frequently to recreational noise at unsafe listening levels [11]. Results of a study that looked at auditory lifestyles and beliefs of college students regarding loud sounds, indicated that 75% of the respondents were aware that exposure to loud sounds can cause hearing loss, and most of them considered hearing loss to be a serious problem [12]. Yet 50% of the students were exposing themselves to loud music, 66% reported experiencing tinnitus, and more than half said that they were not concerned about it since they believed that they would not lose their hearing until a later age. Furthermore, several studies have shown that self-reported PLD users have one or more of the following: hearing loss at one or more frequencies, reduced otoacoustic emissions, and higher incidence of tinnitus [2,4,5,13,14,15,16,17,18]. In addition, ref. [18] showed that subjects listening to higher than 80 dBA had significantly poorer intensity discrimination thresholds, modulation detection thresholds, and syllable identification in noise, supporting cochlear damage in these individuals.

Studies that have exposed subjects in controlled conditions to loud music have also shown similar results. Subjects exposed to 30 min of MP3 player music to an average level of 85 dBC in the ear canal were found to have a significant reduction in distortion product otoacoustic emission (DPOAE) levels in half-octave bands centered from 1.4–6.0 kHz [19]. Young adults exposed to pop-rock music at a full gain setting for one hour had significant changes in hearing thresholds and transient otoacoustic emissions [20]. Additionally, another study [3] that exposed subjects to either rock or pop music at various dBA levels for four hours found TTS for higher levels (greater than 99.5 dBA) of music exposure along with the presence of the notch configuration, mostly at 4 kHz, and reduced DPOAEs in the same frequencies as their TTS. Previously, we [7] showed that music exposure at levels of 100% in as little as 30 min on the Apple iPod Touch 4th Generation resulted in symptoms of overexposure, including tinnitus, TTS, pain, and aural fullness.

Since there are no recommendations or standards for recreational noise exposure, young people continue to expose themselves frequently to unsafe listening levels. Despite the documented risks and consequences of unsafe listening habits, it remains challenging for audiologists to educate, motivate, or persuade the general public effectively to stop the risky behavior and adopt safe listening behaviors. There is a critical need for promoting healthy listening behavior in young adults, and more research is essential to understand the underlying factors that play important roles in listeners’ intentions to engage in safe listening habits.

Various health-related investigations have used a variety of models and theories to study people’s intention to engage in risky behavior. One such model is the Theory of Planned Behavior (TPB) [21]. This theory looks at attitudes, subjective norms and perceived control over the identified behavior, and assesses how these factors influence a person’s behavioral intention to engage in a given behavior. Attitudes are defined as the way that someone feels about performing the specified behavior (positive or negative). Subjective norms are formal or informal rules about how to behave or how the behavior is viewed by others. Perceived behavioral control looks at how much the person feels they have control over doing or not doing a specific behavior in their life. These three factors have been shown to predict behavioral intention and outcomes for a variety of health behaviors ranging from oral health behaviors [22] to alcohol consumption [23].

Previous research has shown TPB to be an effective model of intention in regard to varying behaviors. In 2011, ref. [22] explored the predictive ability of TPB factors on oral hygiene behaviors in young adults and found that attitude and perceived behavioral control, but not subjective norms, contributed towards predicting intention to improve oral health. In their next study in 2013, ref. [24] found that in addition to the above factors, subjective norms and self-identity had a significant correlation with the TPB factors. Several auditory-related studies used TPB to predict the use of hearing protection [25,26,27,28,29,30,31] and found that attitude was a robust predictor of intention to wear hearing protections.

While the above studies shed light on the important factors that might predict one’s use of hearing protection, it is unknown whether the same relationships will hold good for other listening-related behaviors such as decreasing volume or listening for shorter periods of time while listening to loud music. Intensity of exposure and duration of exposure are fundamental factors used in the assessment of noise damage risk criteria and were established for determining acceptable safe and allowable noise levels to prevent noise-induced hearing loss. Regulations that limited allowable levels of occupational noise were based on the physiological response of the ear and allowed for a trade-off between the loudness of sound and the duration of sound [32]. Frequent exposure to loud noise/music for extended periods of time can have a cumulative effect on the auditory system, leading to an increased risk of hearing loss [33]. Hence, in this study, these two risk-controlling variables (decreasing volume and decreasing listening time) were selected as two leading healthy listening behaviors toward reducing the risk of RNIHL.

The barriers to people implementing safe listening habits may conceivably be related to factors such as attitudes and self-control. Nevertheless, it is our belief that the same factors can also be used to overcome or achieve the desired outcomes. Thus, the purpose of this pilot study was to identify attitudes, social norms, and behavioral control in young adults for two PLD-related behaviors: (1) lowering the listening intensity level for loud music, and (2) shortening the duration of time they listen to loud music. We were also interested in assessing whether young adults would exhibit differences in the TPB factors for these two behaviors and if they did, which factors would possibly provide us with a rationale for selecting the appropriate healthy listening communication strategies and potential intervention plans with this population. The long-term goal of our research is to identify strategies for reducing the risk of RNIHL and promote safe listening habits through public education. Decreasing the intensity of music level on their PLDs and decreasing the duration of listening to loud music on their PLD are compensatory ways for people to avoid the potentially damaging effects of RNIHL from PLD usage. Hence, this study was designed to see if attitudes, social norms, and behavioral control can predict intentions to promote safe-listening habits.

## 2. Methods

This study was approved by the university institutional review board (IRB, #18-332). Participants were undergraduate students at the university. All participants read and agreed to a consent form on the first page of the survey before completing the study. Subjects were excluded from survey participation if they were younger than 18 years of age or if they had a known history of hearing loss. The online survey was designed to assess three major factors of the TPB (attitude, perceived behavioral control, and social norms) as they related to participants’ intention to engage in two different behaviors: (1) lowering the listening intensity level for loud music and (2) shortening the duration of time they listen to loud music.

### 2.1. Survey

Students at the university were invited to participate in an online questionnaire/survey that was created using Qualtrics (Qualtrics, Provo, UT, USA). Participants had to first confirm that they (1) are at least 18 years of age, (2) they did not have known hearing loss, and (3) understood that they have to answer all attention questions correctly to ensure that they are involved and paying attention. To assess the study’s main focus, for each of the two behaviors (lowering the listening intensity level for loud music and shortening the duration of time they listen to loud music), participants were asked to respond to survey questions that corresponded with the four key factors of TPB using a seven-point Likert scale (1 = strongly disagree and 7 = strongly agree). Specifically, participants were asked to indicate their perceived social norms surrounding the behavior (six questions), behavioral control (six questions), attitude (eight questions), and intentions to engage in the behavior (six questions) [21,22,26]. Variables of interest were created by averaging across the corresponding survey items. The Cronbach’s alpha values for the variables ranged from 0.64 to 0.89. The participants then completed five demographic questions and questions about previous auditory symptoms following exposure to recreation noise.

### 2.2. Data Analyses

To assess the study’s key research focus, linear regression analyses were performed for each of the two behaviors, with the behavioral intention variable regressed on the three corresponding TPB factors. Following this, paired-sample t-tests were performed to determine whether participants exhibited differences across the TPB factors for the two behaviors of interest.

## 3. Results

### 3.1. Participants

**Figure 1:**
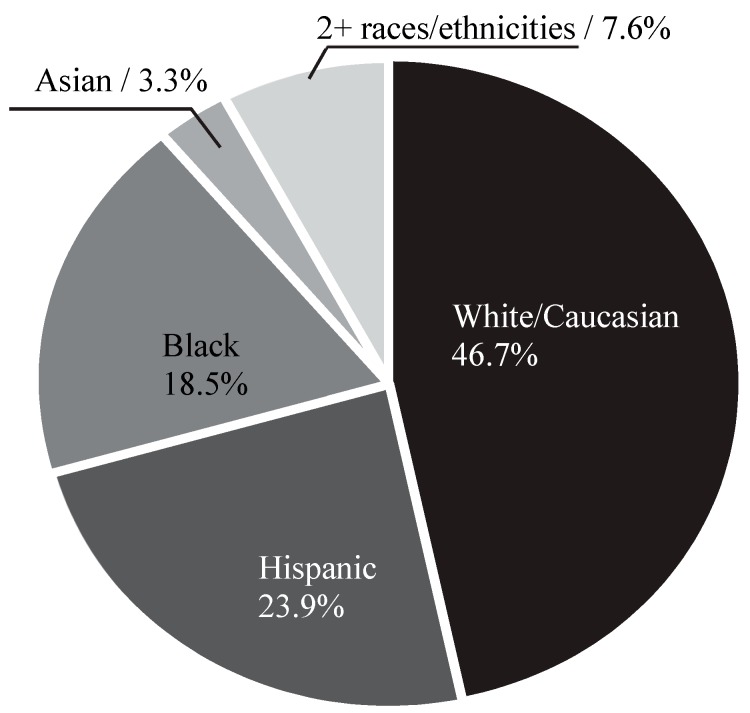
The demographic distribution of race/ethnicity.

A total of 121 undergraduate students opened the survey link. Participants were excluded from further analysis if they did not answer the attention check item correctly or if they did not complete the survey. As such, 92 participants were maintained for analysis. Participants were, on average, 21.60 years of age (SD = 3.41). The sample was diverse in terms of race/ethnicity (Figure 1), ranging from 46.7% of white/Caucasian subjects to 3.3% Asian subjects.

Results from participant reports indicated that about 61% had at least one symptom of RNIHL and 38% had experienced more than one symptom of RNIHL after listening to music through their PLD (Table 1). The most common symptom reported was tinnitus (33%), followed by soreness/ear pain (28%), and limited concentration (22%). Multiple symptoms were reported by 38% of the participants.

### 3.2. Theory of Planned Behavior

**Figure 2:**
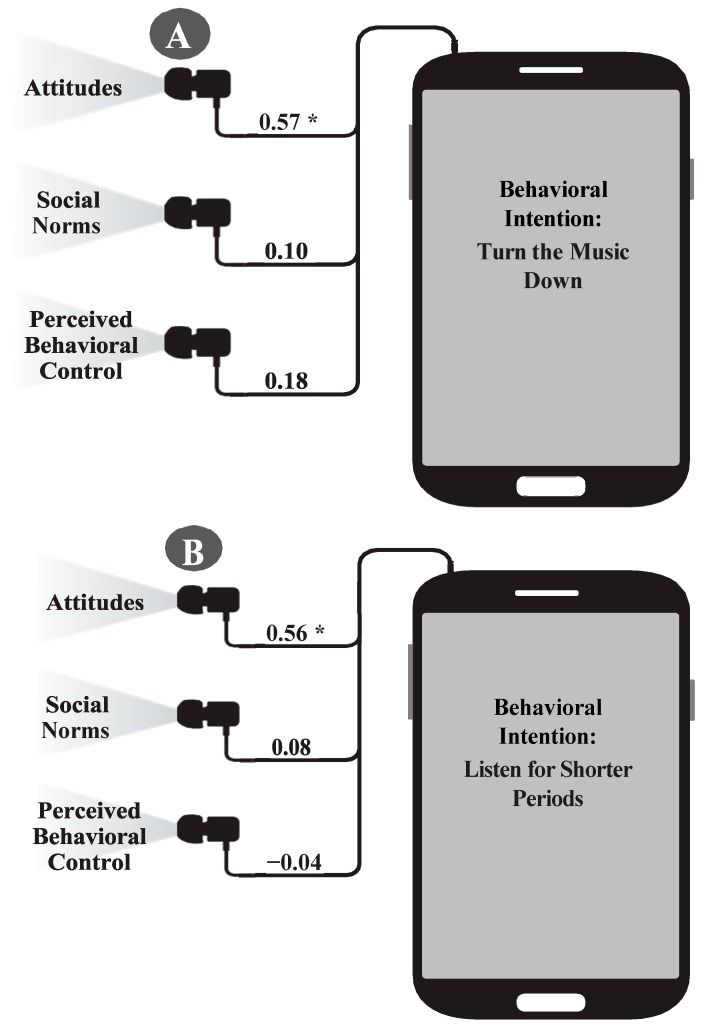
Relationships between attitudes, social norms and perceived behavioral control leading to intention for (**A**) lowering the intensity of loud music and (**B**) shortening duration of loud music. (*) *p* < 0.001.

First, regression analysis indicated that the overall TPB model, wherein behavioral intention to turn the music down was predicted by the three TPB factors (attitudes, social norms, and perceived behavioral control), was significant, *R*^2^ = 0.30, *F* (3, 88) = 12.56, *p* < 0.001. Specifically, a positive attitude toward turning the music down drove intentions to engage in this behavior, *B* = 0.57 (*SE* = 0.12) while the other two predictors, social norms and perceived behavioral control, were found to be non-significant (Figure 2).

Regarding the behavior of listening for shorter durations, the overall TPB model was again significant, *R*^2^ = 0.23, *F* (3, 88) = 8.85, *p* < 0.001. Similar to turning the music down, greater intentions to listen for shorter durations of time were associated with having more positive attitudes toward the behavior, *B* = 0.56 (*SE* = 0.13). Social norms and perceived behavioral control were not found to predict the intention of listening to a PLD for shorter durations of time (Figure 2).

### 3.3. TPB Attitudes

Regression results indicated that the participants’ attitudes toward a given PLD listening behavior played an important role in their intentions to engage in these behaviors. Additionally, it was important to determine whether participants held more positive attitudes toward one behavior over the other. Indeed, it was found that participants had more positive attitudes toward turning loud music down (*M* = 5.81, *SD* = 1.10) than they did toward listening to loud music for a shorter duration (*M* = 5.06, *SD* = 1.32), *t* (91) = 5.91, *p* < 0.001. Means and standard deviations are depicted in Table 2 for both behaviors.

### 3.4. TPB Social Norms

While the regression results indicated that social norms did not play an important role in one’s intentions to engage in healthy PLD listening behaviors, it was important to determine whether participants felt that their social network might support engagement in one behavior over another. Participants indicated that important others (coworkers, friends, and family) were more likely to show support for the participant turning down loud music (*M* = 3.00, *SD* = 1.59) than for the participant listening to music for shorter durations (*M* = 2.63, *SD* = 1.58), *t* (91) = 3.10, *p* < 0.005. However, the overall means for social norms were notably much lower than the overall means of the other TPB factors. Means and standard deviations are depicted in Table 3 for both behaviors.

### 3.5. TPB Perceived Behavioral Control

Participants also indicated that they felt they had more perceived control over turning music on a PLD down (*M* = 6.04, *SD* = 1.07) than they did over listening for shorter durations of time (*M* = 5.52, *SD* = 1.17), *t* (91) = 4.51, *p* < 0.001. Means and standard deviations are depicted in Table 4 for both behaviors.

### 3.6. TPB Intention

The areas of intent involved assessing willingness to engage in behavior, if participants would recommend the behavior to others, and if they would actively look for ways to engage in the behavior. Similar to the TPB factors outlined above, participants also indicated greater intentions for turning the music down (*M* = 5.22, *SD* = 1.37) than listening for shorter periods of time (*M* = 4.11, *SD* = 1.57), *t* (91) = 9.04, *p* < 0.001. The difference in intentions to engage in these two behaviors was considerable (1.11 points) and a much larger difference than between any of the TPB factors. Response averages and standard deviations for this section are shown in Table 5. Results indicated a willingness to engage in both protective behaviors (more so for turning down the volume), but less likely to recommend to others or actively engage in the behaviors.

## 4. Discussion

Exposure to loud music through a PLD is known to have a wide range of damaging short and long-term effects on young adults [2,3,4,5,6,7]. Estimates from WHO suggest that these harmful outcomes are prevalent among young adults worldwide [1]. Therefore, it is important for audiologists to continue to understand how young adults perceive safe listening behaviors and hearing protection actions so that interventions tailored to the needs of this audience can be created. The purpose of the present study was to assess young adult perceptions of two healthy listening behaviors: (1) lowering the listening intensity level for loud music and (2) shortening the duration of time they listen to loud music. These behaviors do not ask young adults to purchase or wear hearing protection, instead, they require preparation or involvement in risk-controlling behavior and may, thus, be an important first step to increasing hearing health safety among this audience. However, previous research has not explored young adults’ perceptions of these two important behaviors.

The present study indicated that of the three TBP factors examined (social norms, attitude, and perceived behavior control), attitude was the only factor that was significantly and positively associated with intent to change behavior. This was true for both safe listening behaviors: lowering the volume on the PLD and reducing the duration of PLD exposure. These results suggest that a more positive attitude toward lowering the music volume level was associated with an increase in intention to turn the music down when it is loud. Similarly, a more positive attitude toward decreasing the duration of music exposure was associated with an increase in intention to listen for shorter periods of time. Thus, attitudes about the listening behaviors seemed to be the determinant of the intentions, rather than social norms or perceived behavioral control.

To add, the study also found that young adults were more inclined to engage in the intentional behavior of lowering the volume level rather than decreasing the duration of PLD usage. This finding is important to consider for future health messaging and intervention work. Given that young adults are more willing to lower the volume, interventions that focus on this behavior might see greater success than those that ask young adults to cut short their listening periods. With this in mind, cell phone applications within popular music-streaming services such as Spotify, Pandora, or Apple Music that signal young adults when the volume is turned up to dangerous levels may be one direction for future work based on the findings from this initial study. Meetings with government and legislation to discuss decibel limitations in bars and clubs may be another avenue for future research.

Additional findings from the study suggested that social norms and perceived behavioral control are not thought to influence young adults’ intentions to engage in the two behaviors of focus. The social norms factor was positively correlated with both safe listening behaviors but did not reach significance. The perceived behavior control factor did not show consistent results across the two listening behaviors. While not formally tested, the overall averages for social norms variables were much lower than the averages for the other TPB factors. These lower values could suggest that young adults feel that individuals who are important to them do not care about their listening behaviors or would not support their decisions to turn the music down or listen for shorter durations. This finding warrants additional investigation. It may be that there are other variables that could explain this finding, such as the presence of stigma or lack of support for positive hearing health behaviors. Health communication campaigns that encourage friends to talk with one another about safe listening might be a way to start the conversation among young adults.

Results of this study are in agreement with several other TBP-related studies that looked at audiological behaviors and non-audiological behaviors [22,25,26,27,28,29,30,31]. In each of these studies, the TPB was implemented to look at behaviors, social norms, attitudes towards noise, use of hearing protection, or hearing conservation strategies in a variety of settings. While results varied across studies, attitudes, either alone or in combination with other factors, were the main predictors of intention for the behaviors identified in each of the studies. Furthermore, intention toward a certain behavior was shown to be significantly associated with the implementation of the behavior in a study which demonstrated that coal miners who intended to wear hearing protection indeed reported actually wearing hearing protection [25].

The findings from this study will guide future research in this area. We believe that large-scale educational approaches to enhance knowledge, attitude, motivation, and self-control are essential to persuade music listeners to follow safe habits in order to reduce their risk for RNIHL. Prevention strategies must be implemented to reduce the risk of RNIHL. As a first step towards this, we will use the findings from this study, where we implemented the TBP model to evaluate the contributing factors towards intention to reduce risky hearing behavior while listening to recreational music. A feasible strategy to reach this group of tech-savvy young adults worldwide is to provide adequate knowledge regarding hearing health through education on social media. Our intents are supported by previous studies, which indicated that providing focused information and knowledge regarding the health care question in hand can significantly affect attitudes towards improved health behaviors among participants [22,28]. Even with the limited sample size, valuable information was obtained regarding the attitudes towards safe listening behaviors in young adults. The data gathered in this study is exploratory in nature and will be used to design future studies that are intended to promote safe listening behaviors. One of the limitations of the study was the relatively small sample size, and the other limitation was the inclusion of only college students. However, the data obtained in this study provided us with the necessary information to launch a larger second survey that is educationally based and message-related, and accessible to the general public.

## 5. Conclusions

This study supported the application of the TPB model to evaluate the factors that may account for the risky behaviors in young adults that can lead to RNIHL. The findings in this study revealed that attitudes have a large influence on young adults’ intention to use safe listening habits for lowering the intensity of loud music and shortening listening periods of loud music on their PLDs. This serves as the foundational study for our future large scale and more in-depth studies that are aimed at using education and persuasion to effect positive attitude in young adults to adopt safer listening habits and thus reduce their risk of hearing loss from recreational noise.

## Figures and Tables

**Table 1:** Percentage of participants reporting previous RNIHL symptoms. Participants selected all of the hearing symptoms they had experienced and could thus select multiple symptoms.

Symptoms	Percentage (%)
Ringing/buzzing in ears	32.6
Ear fullness	17.4
Hearing loss/muffled hearing	9.8
Soreness of ear	28.3
Limited concentration	21.7
Decreased tolerance/annoyance to environmental sounds (hyperacusis)	14.1
More than one symptom	38.0
None of these	39.1

**Table 2:** Means and standard deviations for attitude responses for turning loud music down and listening to loud music for a shorter duration. The overall attitude means for the two behaviors were significantly different, *p* < 0.001. Survey questions used a seven-point Likert scale (1 = strongly disagree and 7 = strongly agree). * reverse coded.

Response	Turning Loud Music Down	Listening to Loud Music for a Shorter Duration
Good	*M* = 5.78 (*SD* = 1.33)	*M* = 5.04 (*SD* = 1.48)
Beneficial	*M* = 6.11 (*SD* = 1.05)	*M* = 5.09 (*SD* = 1.50)
Positive	*M* = 5.64 (*SD* = 1.29)	*M* = 4.90 (*SD* = 1.42)
Negative *	*M* = 5.71 (*SD* = 1.45)	*M* = 5.22 (*SD* = 1.58)
Cronbach’s α	0.86	0.89
Overall	*M* = 5.81 (*SD* = 1.10)	*M* = 5.06 (*SD* = 1.32)

**Table 3:** Means and standard deviations for social norm responses for turning loud music down and listening to loud music for a shorter duration. The overall social norm means for the two behaviors were significantly different, *p* < 0.005. Survey questions used a seven-point Likert scale (1 = strongly disagree and 7 = strongly agree).

Response	Turning Loud Music Down	Listening to Loud Music for a Shorter Duration
**Coworkers**	*M* = 2.54 (*SD* = 1.60)	*M* = 2.45 (*SD* = 1.66)
**Friends**	*M* = 2.74 (*SD* = 1.78)	*M* = 2.48 (*SD* = 1.69)
**Family**	*M* = 3.71 (*SD* = 2.01)	*M* = 2.98 (*SD* = 1.93)
**Cronbach’s α**	0.86	0.88
**Overall**	*M* = 3.00 (*SD* = 1.59)	*M* = 2.63 (*SD* = 1.58)

**Table 4:** Means and standard deviations for perceived behavioral control responses for turning loud music down and listening to loud music for a shorter duration. The overall perceived behavioral control means for the two behaviors were significantly different, *p* < 0.001. Survey questions used a seven-point Likert scale (1 = strongly disagree and 7 = strongly agree). * reverse coded.

Response	Turning Loud Music Down	Listening to Loud Music for a Shorter Duration
Difficult for me *	*M* = 5.57 (*SD* = 1.63)	*M* = 5.05 (*SD* = 1.65)
**No problem**	*M* = 6.08 (*SD* = 1.39)	*M* = 5.38 (*SD* = 1.62)
**Control**	*M* = 6.49 (*SD* = 1.05)	*M* = 6.12 (*SD* = 1.27)
**Cronbach’s α**	0.66	0.64
**Overall**	*M* = 6.04 (*SD* = 1.07)	*M* = 5.52 (*SD* = 1.17)

**Table 5:** Means and standard deviations for behavioral intentions responses for turning loud music down and listening to loud music for a shorter duration. The overall behavioral intention means for the two behaviors were significantly different, *p* < 0.001. Survey questions used a seven-point Likert scale (1 = strongly disagree and 7 = strongly agree).

Response	Turning Loud Music Down	Listening to Loud Music for a Shorter Duration
**Willingness to Engage**	*M* = 6.04 (*SD* = 1.37)	*M* = 4.74 (*SD* = 1.82)
**Recommend to Others**	*M* = 4.62 (*SD* = 1.88)	*M* = 3.79 (*SD* = 1.80)
**Actively Engage**	*M* = 4.99 (*SD* = 1.88)	*M* = 3.79 (*SD* = 1.84)
**Cronbach’s α**	0.68	0.82
**Overall**	*M* = 5.22 (*SD* = 1.37)	*M* = 4.11 (*SD* = 1.57)
